# Proteomics based analysis of the nicotine catabolism in *Paenarthrobacter nicotinovorans* pAO1

**DOI:** 10.1038/s41598-018-34687-y

**Published:** 2018-11-02

**Authors:** Marius Mihăşan, Cornelia Babii, Roshanak Aslebagh, Devika Channaveerappa, Emmalyn Dupree, Costel C. Darie

**Affiliations:** 1 Biochemistry and Molecular Biology Laboratory, Department of Biology, Alexandru Ioan Cuza University of Iaşi, Iaşi, Romania; 2 Biochemistry & Proteomics Group, Department of Chemistry & Biomolecular Science, Clarkson University, Potsdam, NY USA

## Abstract

*Paenarthrobacter nicotinovorans* is a nicotine-degrading microorganism that shows a promising biotechnological potential for the production of compounds with industrial and pharmaceutical importance. Its ability to use nicotine was linked to the presence of the catabolic megaplasmid pAO1. Although extensive work has been performed on the molecular biology of nicotine degradation in this bacterium, only half of the genes putatively involved have been experimentally linked to nicotine. In the current approach, we used nanoLC–MS/MS to identify a total of 801 proteins grouped in 511 non-redundant protein clusters when *P*. *nicotinovorans* was grown on citrate, nicotine and nicotine and citrate as the only carbon sources. The differences in protein abundance showed that deamination is preferred when citrate is present. Several putative genes from the pAO1 megaplasmid have been shown to have a nicotine-dependent expression, including a hypothetical polyketide cyclase. We hypothesize that the enzyme would hydrolyze the N1-C6 bond from the pyridine ring with the formation of α-keto- glutaramate. Two chromosomally-encoded proteins, a malate dehydrogenase, and a D-3-phosphoglycerate dehydrogenase were shown to be strongly up-regulated when nicotine was the sole carbon source and could be related to the production the α-keto-glutarate. The data have been deposited to the ProteomeXchange with identifier PXD008756.

## Introduction

Nicotine is the main alkaloid produced by the tobacco plant as an anti-herbivore chemical. Due to its potent parasympathomimetic stimulant effect, nicotine plays a crucial role in smoking addiction and proved to be toxic in high concentrations to both animals and humans^1^. Nicotine can accumulate as both liquid and solid tobacco waste during the manufacturing of tobacco products. As it is water soluble, nicotine can easily contaminate the environment and endanger human health and aquatic life.

Several bacterial strains have been shown to use nicotine as their sole carbon and nitrogen source for growth. Generally referred as Nicotine-Degrading Microorganisms (NDMs)^2^, these bacteria offer an eco-friendly method for removing nicotine from tobacco products and waste by means of bioremediation. The NDMs could also provide enzymes and pathways that can be engineered to convert nicotine and other pyridine and/or pyrrolidine ring containing compounds into starting materials for the synthesis of products of industrial and pharmaceutical importance. The development of technologies for converting nicotine waste into 6-Hydroxy-3-succinoyl-pyridine^3^, 3-succinoyl-pyridine^4^ or 6-hydroxy-nicotine^5^ as well as the recent identification of 6-hydroxy-nicotine as a neuroprotective drug^6^ shows that NDMs nicotine pathways and enzymes have valuable biotechnological applications and could be used for the production of green chemicals.

**Figure 1:**
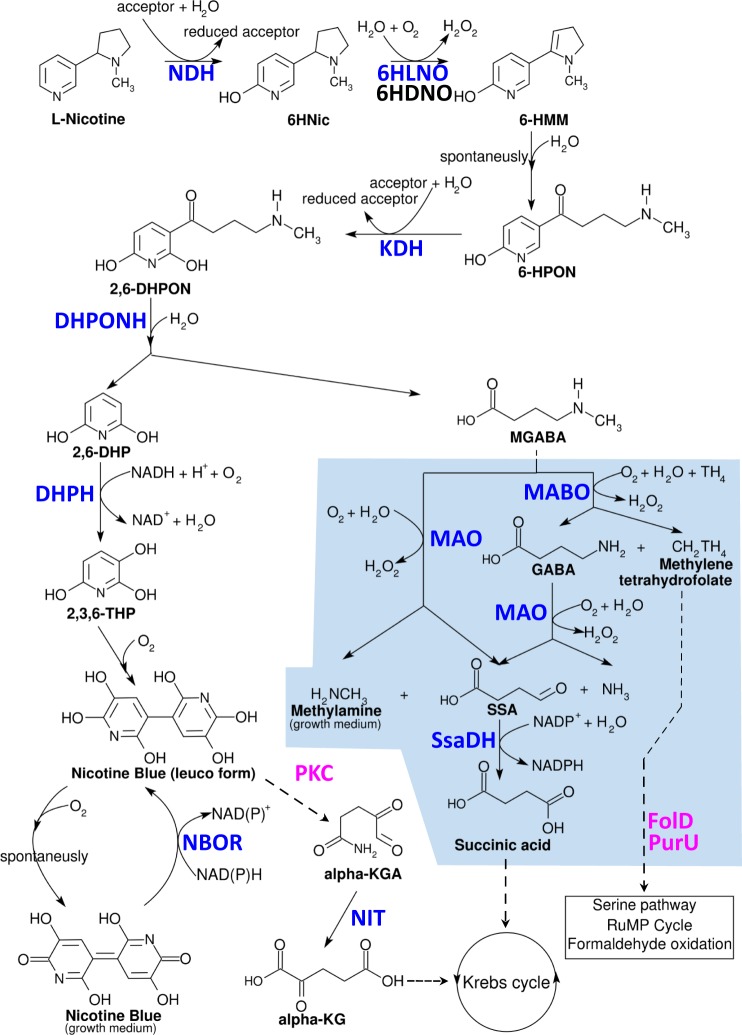
Overview of the nicotine catabolic pathway of *Paenarthrobacter nicotinovorans* and the pAO1 encoded enzymes identified in the current proteomics experiment. Blue text indicates enzymes shown to have a nicotine-dependent expression by the proteomics experiment. Magenta text indicates ORF’s with putative function related to nicotine and shown to have a nicotine-dependent expression by our proteomics experiment. Black text indicates enzymes that were not detected as expressed in our proteomics experiment. Light blue background indicates the lower nicotine pathway. CAPS indicate enzymes catalyzing the stepwise degradation of nicotine: NDH - nicotine dehydrogenase; 6HLNO - 6-hydroxy-L-nicotine oxidase; 6HDNO - 6-hydroxy-D-nicotine oxidase; KDH - ketone dehydrogenase; DHPONH - 2,6-dihydroxypseudooxynicotine hydrolase; DHPH – 2,6-dihydroxypyridine-3-hydroxylase NBOR – nicotine blue oxidoreductase; MABO - γ-N-methylaminobutyrate oxidase; FolD - methylene-tetrahydrofolate dehydrogenase/cyclohydrolase; PurU - formyl-tetrahydrofolate deformylase; MAO - monoamine-oxidase; SsaDH - succinic semialdehyde dehydrogenase; PKC – putative polyketide cyclase; NIT - ω-amidase. CAPS AND BOLD letters indicate the intermediates: 6HNic – 6-hydroxynicotine; 6-HMM – 6-hydroxy-methylmyosmine; 6-HPON – 6-hydroxy-pseudooxynicotine; 2,6-DHPON – 2,6-dihydroxypseudooxynicotine; 2,6-DHP – 6-dihydoxypyridine; CH3-4-GABA - γ-N-methylaminobutyrate; 2,3,6-THP - 2,3,6-trihydroxypyridine; NB -nicotine blue, 4,4′,5,5′-tetrahydroxy-3,3′-diazadiphenoquinone-(2,2′); CH2 TH4 - methylenetetrahydrofolate; GABA - γ-aminobutyric acid; SSA - succinic semialdehyde, alpha-KGA - a-keto-glutaramate; alpha-KG - a-keto-glutarate.

*Paenarthrobacter nicotinovorans* is a soil Gram-positive NDM that first came into the limelight in the 1960’s under the name *Arthrobacter oxidans*^7^, re-classified as *Arthrobacter nicotinovorans*^8^ in the 1990’s and received its current name in 2016^9^. *P*. *nicotinovorans* ability to use nicotine as carbon and energy source is linked to the presence of a cluster of nicotine-inducible genes (*nic*- genes) placed on the catabolic megaplasmid pAO1^10^. *P*. *nicotinovorans* makes use of the pyridine pathway to degrade nicotine. Briefly, nicotine catabolism starts with hydroxylation of the pyridine ring and results in formation of γ-N-methylaminobutyrate (CH_3_-4-GABA) and 2,6-dihydroxy-pyridine (2,6-DHP). CH_3_-4-GABA is degraded to succinate and methylamine in what is called the lower nicotine pathway, the latter compound been shown to accumulate into the growth medium^11^. 2,6-DHP is hydroxylated to trihydroxy- pyridine (THP) which spontaneously dimerizes to 4,4′,5,5′-tetrahydroxy-3,3′-diazadiphenoquinone-(2,2′) (Nicotine-blue, NB) giving the characteristic nicotine-blue color (for an overview see reference^12^, Fig. 1 and supplementary Fig. 1).

The remarkable work performed by the Decker and later the Brandsch group on the molecular biology and biochemistry of nicotine degradation in *P*. *nicotinovorans*^13^ lead to the experimentally establishment of functions for 19 out of the 40 genes making out the *nic*-gene cluster^12^. The rest of the *nic* genes either have putative or no known functions or are may not be expressed at all. These genes might include some missing key points in nicotine metabolism such as major gene regulators or nicotine transporters. An experimental indication of the involvement of these putative genes in the nicotine metabolism is required.

The current shotgun proteomics approach attempts to fill this gap by identifying the proteins expressed by *P*. *nicotinovorans* in the presence of nicotine using nanoLC–MS/MS. A total of 801 proteins grouped in 511 non-redundant protein clusters were identified when *P*. *nicotinovorans* was grown on citrate, nicotine and citrate, and nicotine as the only carbon source. The differences in protein expression patterns allowed us not only to fill in some of the blank spots in nicotine metabolism, but also to relate this plasmid-encoded catabolic pathway to the general chromosome-encoded pathways of the cell. This proteomics data can provide some valuable insight into the genetics and physiology of nicotine metabolism and serve as a basis for generating hypotheses for future attempts to genetically-engineer the catabolic pathway for increased bioremediation efficiency or production of green chemicals.

## Results and Discussion

### Proteins identification overview

The cell free lysates from *Paenarthrobacter nicotinovorans* pOA1 growing on citrate, nicotine and citrate and nicotine as the only carbon sources were first separated by sodium dodecyl sulfate-polyacrylamine gel elctrophoresis (SDS-PAGE), then digested with trypsin and the resulting peptides analyzed by nanoLiquid Chromatography Tandem Mass Spectrometry (nanoLC-MS/MS). A bottom-up approach based on the MASCOT MS/MS database search algorithm was used to identify the proteins in each sample. Currently, there is no final genome of *P*. *nicotinovorans* pAO1 available, so for the database searches a custom database was used. The database included the reference proteome of *P*. *aurescens* strain TC1, the complete proteome of pAO1 megaplasmid as well as the partial proteome derived from the draft genome of *P*. *nicotinovorans* strain Hce-1 (WGS sequence set BDDW01000000). This approach allowed us to identify a total of 801 proteins grouped in 511 non-redundant protein clusters. The full list of proteins identified in this study is presented in Supplementary Table 1. The distribution of these clusters showing the overlap between the proteomes of *P*. *aureus* TC1 and *P*. *nicotinovorans* is depicted in Table 1. A total number of 368 non-redundant proteins were identified based on genes present in both the *P*. *aureus* TC1 and the *P*. *nicotinovorans* making out the core-genome of the two species. Moreover, a number 115 non-redundant proteins hits were identified based on genes that are only found in the *P*. *nicotinovorans* pangenome, 28 being encoded by the pAO1 megaplasmid. One unexpected finding is that a number of 30 non-redundant proteins hits were identified based on genes that are specific for *P*. *aureus* TC1 genome. This can only be explained by the fact that the WGS sequence set BDDW01000000 is not the complete genes set for the *P*. *nicotinovorans* strain.

**Table 1:** Distribution of the shared and specific non-redundant proteins identified in the cell free lysates of *Paenarthrobacter nicotinovorans* pAO1.

Proteome	Dimension (sequences)	Proteins			Total number of proteins
		Citrate	Nicotine	Nicotine and Citrate	
*Paenarthrobacter aureus* TC1	4565	332	306	218	397
*Paenarthrobacter nicotinovorans*	4722	377	369	268	482
Specific to *P*. *nicotinovorans*	—	66	85	62	115
pAO1 megaplasmid	175	1	21	27	28
Total number of non-redundant proteins	—	398	391	279	511

**Figure 2:**
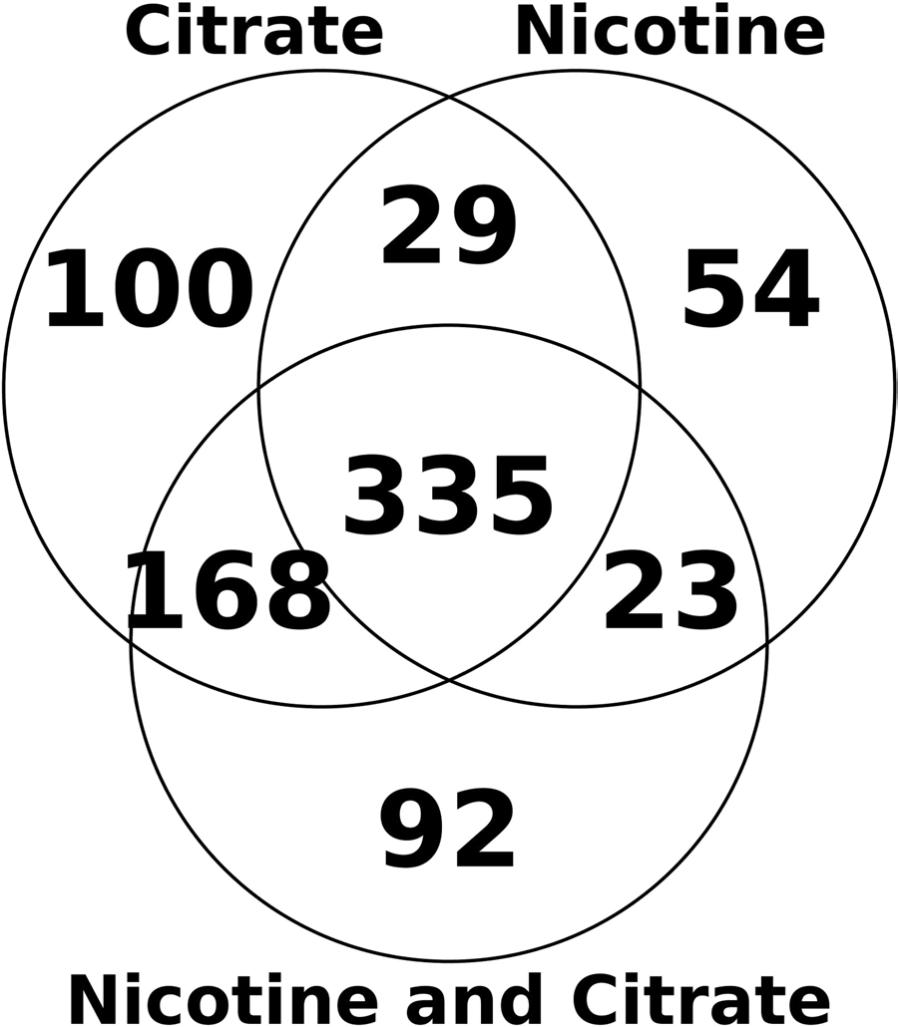
Venn diagram illustrating overlaps between the substrate specific proteins identified by LC-MS/MS analysis of *Paenarthrobacter nicotinovorans* pAO1 grown of different carbon sources.

**Figure 3:**
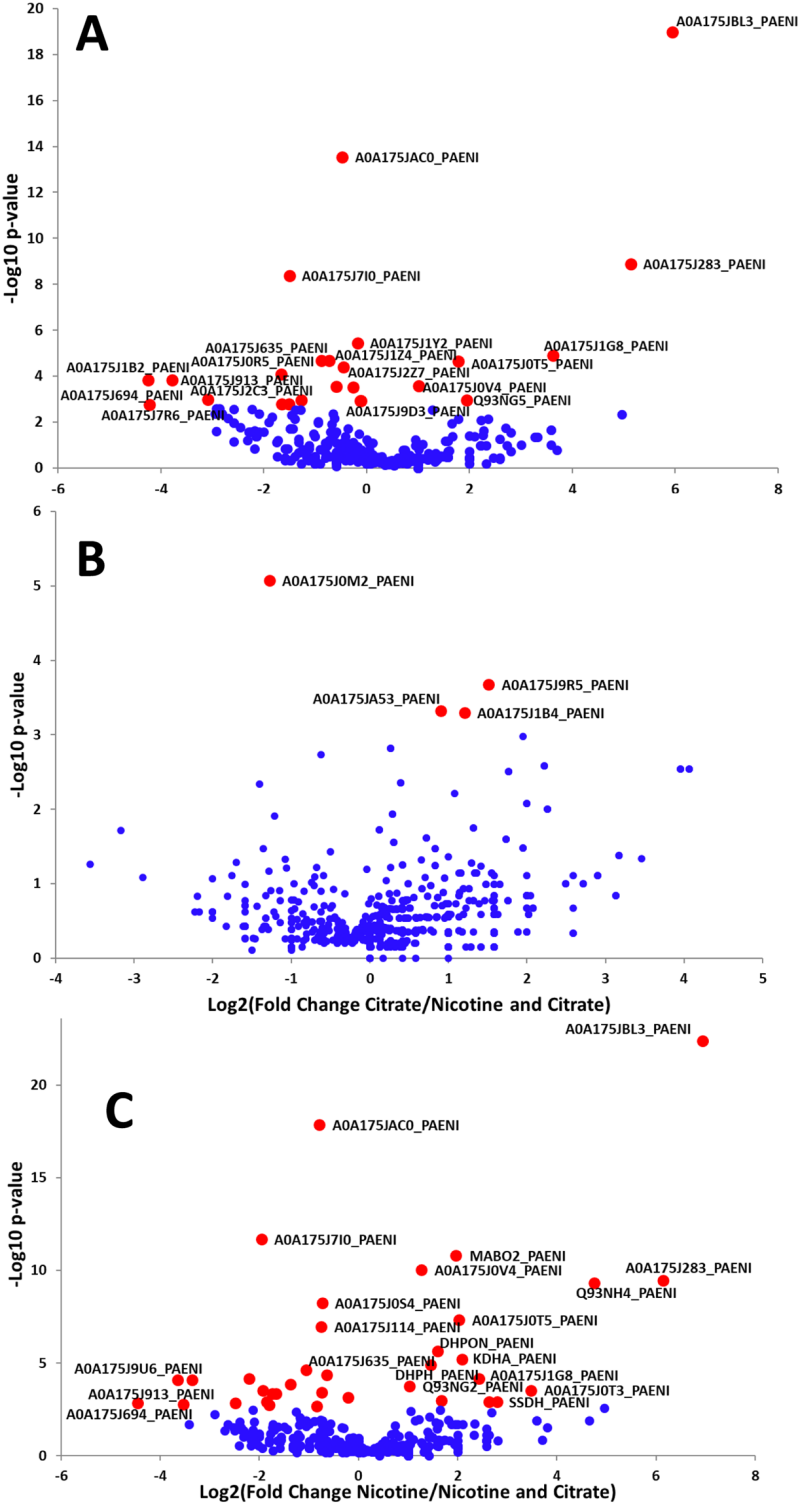
Volcano plots demonstrating the magnitude (x-axis) and significance (y-axis) of the protein comparisons between: (**A**) Nicotine *versus* Citrate, (**B**) Citrate *versus* Nicotine and Citrate and (**C**) Nicotine *versus* Nicotine and Citrate. Red dots represent significantly altered proteins (p < 0.001), whereas blue dots represent statistically insignificant proteins. The labels indicate the UniProKB IDs for the proteins listed in Supplementary Table 2.

The overlap between the shared and unique proteins expressed in *P*. *nicotinovorans* pAO1 cells growing on different carbon sources is summarized in the Venn diagram from Fig. 2. Disregarding whether it is used as the sole Carbon source, the presence of nicotine in the growth medium triggers several important changes in the protein expression patterns of *P*. *nicotinovorans*. The magnitude and significance of these changes can be observed in the Volcano plots depicted in Fig. 3. Due to the specific way the Scaffold software calculates the fold change, the proteins not expressed in any of the compared conditions do not show up on the graphs. In the cells grown solely on nicotine, 46 out of 632 identified proteins were dysregulated when compared to the citrate grown nicotine cells. When both nicotine and citrate were present as the carbon source, the number of dysregulated proteins was only 16 out of 618. The full list of statistically significant dysregulated proteins in all growth conditions is presented in Supplementary Table 2.

### Differentially regulated plasmid-encoded proteins in *P*. *nicotinovorans* in the presence of nicotine

As expected, none of the proteins encoded by the pAO1 megaplasmid and known to be involved in nicotine metabolism could be identified in the cell free extracts from the cells grown on citrate alone. The presence of nicotine in the growth media alone or in combination with citrate leads to the expression of all the pAO1 enzymes known to be responsible for converting nicotine to γ-N-methylaminobutirate: nicotine dehydrogenase (NDH), 6-hydroxy-L-nicotine oxidase (6HLNO), 6-hydroxypseudooxynicotine dehydrogenase (KDH) and 2,6-dihydropseudooxynicotine hydrolase (DHPONH). Also, the enzymes responsible for the production of nicotine blue, the 2,6-dihydroxypyridine 3-monooxygenase (DHPH) and the NAD(P)H-Nicotine Blue Oxidoreductase (NBOR) were also identified when nicotine was present in the growth medium (Table 2).

**Table 2:** Differentially regulated pAO1-encoded proteins during the growth of *Paenarthrobacter nicotinovorans* on different carbon sources.

	Protein	UniProt ID	Compared growth conditions					
			Nicotine *vs* Citrate		Nicotine and Citrate *vs* Citrate		Nicotine *vs* Nicotine and Citrate	
			Fold Change	Fisher’s exact test (p < 0.00245)	Fold Change	Fisher’s exact test (p < 0.00077)	Fold Change	Fisher’s exact test (p < 0.00249)
1	**2,6-dihydropseudooxynicotine hydrolase, DHPONH**	DHPON_PAENI	INF	<0.00010	INF	<0.00010	3	<0.00010
2	**2,6-dihydroxypyridine 3-monooxygenase, DHPH**	DHPH_PAENI	INF	<0.00010	INF	<0.00010	2.8	<0.00010
3	**2-oxoglutaramate amidase, NIT**	NIT_PAENI	INF	<0.00010	INF	<0.00010	2.3	0.011
4	**4-methylaminobutanoate oxidase (formaldehyde-forming), MABO**	MABO1_PAENI	INF	0.00013	n.d	—	INF	<0.00010
5	**4-methylaminobutanoate oxidase (methylamine-forming), MAO**	MABO2_PAENI	INF	<0.00010	INF	<0.00010	3.9	<0.00010
6	**6-hydroxy-L-nicotine oxidase 6-HLNO**	Q93NH4_PAENI	INF	<0.00010	INF	0.00029	27	<0.00010
7	**6-hydroxypseudooxynicotine dehydrogenase complex subunit alpha, KDHA**	KDHA_PAENI	INF	<0.00010	INF	<0.00010	4.2	<0.00010
8	Bifunctional protein FolD	Q8GAI4_PAENI	INF	0.00069	n.d	—	INF	0.0002
9	Formyltetrahydrofolate deformylase, PurU	Q8GAI2_PAENI	INF	0.0017	n.d	—	INF	0.0014
10	Hypothetical polyketide cyclase, PKC	Q93NG4_PAENI	INF	<0.00010	INF	<0.00010	1.4	0.15
11	**NAD(P)H-Nicotine Blue (4,4′,5,5′-tetrahydroxy-3,3′-diazadiphenoquinone-(2,2′)) Oxidoreductase, NBOR**	Q8GAJ2_PAENI	INF	<0.00010	INF	<0.00010	1.4	0.23
12	**Nicotine dehydrogenase large subunit NDHL**	Q93NH5_PAENI	INF	<0.00010	INF	0.00031	3.1	0.0058
13	**Nicotine dehydrogenase medium subunit, NDHM**	Q59127_PAENI	INF	<0.00010	INF	<0.00010	2.7	0.0078
14	**Nicotine dehydrogenase small subunit, NDHS**	Q59129_PAENI	INF	<0.00010	INF	0.00031	3.1	0.0058
15	ORF106, putative molybdenum transport ATPase, MODC	Q93NG5_PAENI	INF	0.0011	INF	<0.00010	1.5	0.25
16	ORF78, putative carbon monoxide dehydrogenase subunit G, COXG	Q93NG2_PAENI	INF	<0.00010	INF	<0.00010	2	0.00017
17	ORF76, putative carbon monoxide dehydrogenase subunit D, COXD	Q93NG0_PAENI	INF	<0.00010	INF	<0.00010	1.5	0.24
18	**Succinate-semialdehyde dehydrogenase SsaDH**	SSDH_PAENI	INF	<0.00010	INF	0.00023	7.4	0.00013
19	ORF92, hypothetical DUF948 domain-containing protein	Q8GAH6_PAENI	INF	0.00032	n.d	—	INF	0.00014

^a^Fold Change as calculated by Scaffold v.4.8.2– weighted number of spectral counts for one protein in one condition vs weighted number of spectral counts in the second condition; a value is defined “significant” when it is either greater than or equal to 2.0 (protein is upregulated) or it is less than or equal to 0.5 (protein is down-regulated). INF - protein identified in the first condition, but not second, 0 - Protein identified in the second condition, but not first, n.d. - protein not detected in any of the compared conditions.

Bold typeface indicates proteins that have been shown experimentally to be involved in nicotine catabolism.

### Deamination is preferred in the lower nicotine pathway when citrate can be used as a Carbon source

The lower nicotine pathway of *P*. *nicotinovorans*, consists of either a deamination or demethylation of γ-N-methylaminobutyrate (CH_3_-4-GABA)^11^ resulted from the pyrrolidine ring cleavage. As shown in Fig. 1, in the deamination pathway, CH_3_-4-GABA is converted by MAO, a methylamine-forming 4-methylaminobutanoate oxidase (MABO2_PAENI) to succinic-semialdehyde. The methylamine is excreted in the growth medium^14^, while the succinic-semialdehyde is further converted by the downstream enzyme succinic-semialdehide dehydrogenase SsaDH (SSDH_PAENI) to succinic acid and integrated into the tricarboxylic acid (TCA) cycle^11^. In the demethylation pathway, CH_3_-4-GABA is converted by MABO, a formaldehyde-forming 4-methylaminobutanoate oxidase (MABO1_PAENI) in γ-aminobutyrate (GABA), methylenetetrahydrofolate (CH_2_-THF) and reduced FADH_2_^15^. By using revers transcription PCR, Chiribau *et al*.^11^ showed the nicotine dependent expression of both *mao*, *ssdh* and *mao* genes and postulated that the both pathways must be active *in-vivo*. Using *in vitro* kinetic data, the authors also predicted a preferentially channeling of CH_3_-4-GABA to the demethylation pathway, despite the fact that methylamine was known to accumulate in the growth medium.

Our MS/MS data showed an unexpected finding: the lower nicotine pathway enzymes of *P*. *nicotinovorans* are differently expressed based on the available C sources. The MAO and SsaDH enzymes were identified in both the nicotine, and nicotine and citrate containing media, thus explaining the previously reported accumulation of methylamine. MABO, the key enzyme in the demethylation pathway, was found only when nicotine alone was used as a carbon source. Moreover, *folD* and *purU*, two putative genes located on the same pAO1 encoded operon as the gene encoding ABO, are also expressed when nicotine is present, but citrate is absent. The *folD* gene encodes a bifunctional protein (Q8GAI4_PAENI) putatively involved in the oxidation of CH_2_-THF to 10-formyltetrahydrofolate (10-CHO-THF). The *purU* gene encodes a formyltetrahydrofolate deformylase (Q8GAI2_PAENI) that catalyzes the hydrolysis of 10-CHO-THF to formate and tetrahydrofolate (THF). The sequence of enzymes MABO, FolD, PurU will thereby provide a way of transforming the methyl group from CH_3_-4-GABA in formaldehyde that can be assimilated by the RuMP cycle, serine pathway or directly by formaldehyde oxidation^16^.

Apparently, when both nicotine and citrate can be used as a C source, *P*. *nicotinovorans* uses the deamination pathway to quickly extract the succinic acid from CH_3_-4-GABA and to use it in the TCA cycle. When carbon sources are scarce and only nicotine is available, the demethylation pathway is activated in *P*. *nicotinovorans*. This generates GABA that can be converted to succinic acid and used again in the TCA cycle, but has the advantage of generating one extra methyl group that can be used for the synthesis of sugars or amino acids.

### Experimental evidence of a hypothetical polyketide cyclase involvement in nicotine metabolism

Another interesting finding is that a hypothetical polyketide cyclase (PKC, Q93NG4_PAENI) has a nicotine-dependent expression (Table 2). BLAST searches using the PKC amino acid sequence gave several domain hits: a specific hit for pfam04199 (putative cyclases, E-value = 9.71e-30) and two non-specific hits for COG1878 (kynurenine formamidase, E-value = 7.58e-29) and TIGR03035 (arylformamidase, E-value = 6.14e-05). The bacterial kynurenine formamidase are metal-dependent hydrolases^17^ acting on carbon-nitrogen bonds other than peptide bonds. As the *pkc* is apparently placed in the same operon with *kdhL* and *dhph* encoding the key enzymes for the production of 2,3,6-THP (Fig. 3), it is highly tempting to hypothesize that PKC would hydrolyze the N1-C6 bond in 2,3,6-THP with the formation of α-keto- glutaramate. An indirect indication that this might be the case is the fact that the 2-oxoglutaramate amidase (NIT, NIT_PAENI) is also expressed only when nicotine is present in the medium (Table 2). The NIT activity converting α-keto-glutaramate to α-keto-glutarate was experimentally shown and it was postulated that the enzyme connects the nicotine pathway to the citric acid cycle and makes the pyridine nitrogen available for assimilation via a glutamate dehydrogenase^18^. The 2-Hydroxypyridine degradation pathway in *Rhodococcus rhodochrous* strain PY11 also has THP as an intermediate connects and to the citric acid cycle in a very similar manner^19^. It makes use of HpoH, a THP hydroxylase and HpoI, a 2-ketoglutaramate amidase. HpoH of *R*. *rhodochrous* PY11 and PKC of *P*. *nicotinovorans* pAO1 share a level of 77% sequence similarity and the two amidases share 47% sequence similarity.

### Differentially regulated chromosomally-encoded proteins in *P*. *nicotinovorans* in the presence of nicotine

A striking fold change related to nicotine was recorded for two chromosomally-encoded enzymes: a malate dehydrogenase (A0A175JBL3_PAENI) and a D-3-phosphoglycerate dehydrogenase (PHGDH, A0A175J283_PAENI). In both cases, the enzymes are up-regulated when nicotine is the sole carbon source present in the medium but are not significantly dysregulated when both nicotine and citrate are present. In addition to catalyzing oxidation of 3-phosphoglycerate, PHGDH has been shown to catalyze the NADH-dependent reduction of α-keto-glutarate^20^ and its high expression would fit very well with the above mentioned expression of NIT and production of α-keto-glutarate from the pyridine ring of nicotine. The malate dehydrogenase is involved in the oxidation of malate to oxaloacetate in many metabolic pathways, including the citric acid cycle. The high expression levels of these two enzymes must be related to the cells effort to concentrate all the available carbon derived from nicotine metabolism into the major metabolic pathways of the cell required for growth. When citrate is abundant in the medium (alone or in combination with nicotine), such an effort is not required and the two enzymes return to a more basal expression.

Another general metabolism enzyme that can be linked to the presence of nicotine in the growth medium is a catalase (A0A175J0M2_PAENI). The enzyme has approx. 2.5-fold change when nicotine is present in the growth medium, disregarding weather citrate is present or not. Interestingly, the expression levels on nicotine only and nicotine and citrate media are the same. The production of nicotine-blue pigment involves a spontaneous oxidation reaction accompanied by the release of O_2_^−^. It was postulated that this oxidation reaction may represent a selective advantage for *P*. *nicotinovorans* bacteria in competition with other soil community bacteria sensitive to oxygen radicals^21^ and that the NAD(P)H-Nicotine Blue Oxidoreductase (NBOR) enzyme might prevent the formation of these radicals inside the cell. Our MS data indicate that the mechanism by which *P*. *nicotinovorans* itself appears not to be affected by the generation of these radicals during growth on nicotine is the increased expression of catalase and that the role of the NBOR must be reconsidered. Considering the MS/MS data showing the nicotine dependent expression of PKC and NIT, we can assume that Nicotine-blue reduction reaction catalyzed by the NBOR is actually preparing the pyridine ring to be cleaved by PKC. The product of the cleavage reaction is then converted to α-keto-glutarate by the plasmid –encoded amidase NIT and integrated into the general metabolism by the chromosomally-encoded PHGDH.

In conclusion, we used nanoLC–MS/MS to identify a total of 801 proteins grouped in 511 non-redundant protein clusters when *P*. *nicotinovorans* was grown on different C-sources. The differences in protein abundance showed that deamination is preferred in the lower nicotine pathway when citrate is present in the medium. A hypothetical polyketide cyclase was shown to have a nicotine-dependent expression and we hypothesize that the enzyme would hydrolyze the N1-C6 bond from the pyridine ring with the formation of α-keto- glutaramate. Two chromosomally-encoded proteins, a malate dehydrogenase, and a D-3-phosphoglycerate dehydrogenase were shown to be strongly up-regulated when nicotine was the sole carbon source and could be related to the production the α-keto-glutarate. The proteomics data have been deposited to the ProteomeXchange with identifier PXD008756 and might serve as a basis for generating hypotheses for future attempts to genetically-engineer the catabolic pathway for increased bioremediation efficiency or production of green chemicals.

## Materials and Methods

### Materials

All materials were purchased from Sigma-Aldrich (St. Louis, MO, US) if not stated otherwise. DNase 1 and RNase A were from Roche (Basel, Switzerland). HPLC grade water and Acetonitrile were from Fisher Chemical (Pittsburgh, PA, USA). LC-MS grade formic acid was from Fluka (Buchs, Switzerland) and Iodoacetamide from Calbiochem.

### Bacterial strain and growing conditions

*Paenarthrobacter nicotinovorans* pAO1 *(*DSM 420 *Deutsche Sammlung von Mikroorganismen und Zellkulturen*, Braunschweig, Germany) was grown on 34 mM Na_2_HPO_4_-22 mM KH_2_PO_4_ buffer, pH 7.0, 0.2% (NH_4_)_2_SO_4_, supplemented with 5% mineral solution, 0.1 mg ml^−1^ biotin and 35 ug ml^−1^ kanamycin. As the carbon source 0.05% nicotine, 0.2% sodium citrate or the combination of the two were used. Bacterial cultures were incubated at 28 °C on a rotary shaker (Model 3013, GFL, Burgwedel, Germany) at 180 rpm for 10 hours until nicotine was depleted from the medium and the specific nicotine-blue color is formed.

### Cell-lysis and sample preparation

Bacteria were collected by centrifugation at 4,500 × g, 20 min from cultures grown in the presence or absence of nicotine; the bacterial pellets were washed twice in 10 mM TRIS/HCl pH 7.4 for the removal of the nicotine-blue dye. Cells were lysed according to the protocol of Vandera *et al*.^22^, with slight modifications. 0.5 g of cells were resuspended in 5 ml 40 mM Tris–HCl, pH 7.6, 0.03% PMSF (w/v), 0.005% chloramphenicol (w/v), 18% sucrose (w/v) in the presence of 50 mg ml^−1^ lysozyme and incubated for 60 min at 37 °C. After centrifugation, cell pellets were washed with 10 mM Tris–HCl, pH 7.6 and resuspended in 5 ml lysis buffer containing 40 mM Tris–HCl, pH 7.6, 0.3% SDS (w/v), 60 mM DTT, 10 μg ml^−1^ DNase I and 10 μg ml-1 RNase A. Samples were incubated for 30 min at 95 °C with intervals of vigorous vortexing every 5 min and then centrifuged on an Eppendorf 5417R centrifuge at 14,000 g and 4 °C for 30 minutes. The cell lysates were quickly placed on ice and stored at −20 °C until further processing. Protein concentrations were determined by the BCA method, using an assay kit (Sigma Aldrich, Germany) with bovine serum albumin (BSA) as a standard.

One hundred μg of total proteins from the cell free lysates were separated on a home-made 9–16% SDS-PAGE maxi (20 × 20 cm) gradient gels and then stained by Commasie Brilliant Blue R250. Each biological sample was loaded in triplicate. The gel lanes for different biological samples were divided into 20 gel pieces and then subjected to in-gel digestion using trypsin, as described previously^23^. Briefly, each gel piece was washed under moderate shaking at room temperature (RT) in HPLC grade water for 60 min and then in 50% (v/v) acetonitrile (ACN)/HPLC grade water containing 50 mM ammonium bicarbonate (ABC) for 60 min. After a dehydration step with 100% ACN for 60 min, the gel pieces were dried under Speed Vac and the Cys residues were further reduced and alkylated using with 10 mM dithiothreitol (DTT) in 25 mM ABC for 60 min. at 60 °C and with 100 mM iodoacetamide in 25 mM ABC for 60 min. in the dark. Gel pieces were dehydrated and dried again. The digestion was performed by rehydrating the gel pieces in 200 μl of a trypsin solution (10 ng/μl) and incubating overnight at 37 °C. After incubation, peptide extraction was carried out with 5% formic acid (FA) in 50/50 (v/v) 50 mM ABC/ACN and with 5% FA in ACN (60 min. each). Extracted peptides were dried and then cleaned by reversed-phase chromatography using C18 ZipTips (EMD Millipore, Billerica, MA).

### Nanoliquid chromatography tandem mass spectrometry (nanoLC-MS/MS)

The resulting peptide mixture was analysed by reversed phase nanoLiquid Chromatography Tandem Mass Spectrometry (nanoLC-MS/MS) using a NanoAcquity UPLC (Waters, Milford, MA, USA) coupled to a Q-TOF Xevo G2 MS (Waters), according to published procedures^24,25^. The peptides were loaded onto a 100 μm × 10 mm NanoAquity BEH130 C18 1.7 μm UPLC column (Waters) and eluted over a 180-min. gradient at a flow rate of 400 nL/min as follows: 1–45% organic solvent B (ACN containing 0.1% FA) over 1–120 min., 45–85% B (120–140 min.), constant 85% B (140–160 min.), 85%–2% B (160–165 min.) and then return to the initial conditions of 1% B (165–180 min.). The aqueous solvent A was 0.1% FA in HPLC water. The column was coupled to a Picotip Emitter Silicatip nano-electrospray needle (New Objective, Woburn, MA, USA). MS data acquisition involved survey 0.5 sec. MS scans (m/z range 350–2000) and automatic data dependent analysis (DDA) of the top six ions with the highest intensity, with the charge of 2+, 3+ or 4+. The MS/MS (recorded over m/z of 50–2000) was triggered when the MS signal intensity exceeded 500 counts/sec. In survey MS scans, the six most intense peaks were selected for collision-induced dissociation (CID) and fragmented until the total MS/MS ion counts reached 6000 or for up to 1.1 sec. each. The entire procedure used was previously described^24^. Ion intensity was adjusted using Leucine enkephalin (2 ng ul^−1^) in direct infusion ESI-MS at 0.5 μL/min. Calibration was performed for both precursor and product ions using direct infusion ESI-MS at 0.5 μL/min of 1 pmole GluFib (Glu1-Fibrinopeptide B) standard peptide with the sequence EGVNDNEEGFFSAR and the theoretical monoisotopic (2+) peak with m/z of 785.84.

### Data analysis

The raw files were analyzed using ProteinLynx Global Server v.2.4 (Waters, Milford, MA, USA) and Mascot v.2.5.1 (Matrix Science, London, UK). Peak list files were generated using the following parameters: background subtraction of polynomial order five adaptive with a threshold of 30%, two smoothings with a window of three channels in Savitzky-Golay mode and centroid calculation of top 80% of peaks based on a minimum peak width of four channels at half height. Database searches were performed using a customized database containing the complete reference proteome of *Paenarthrobacter aurescens* strain TC1 (UP000000637)^26^, the proteome of *Paenarthrobacter nicotinovorans* strain Hce-1 (UP000078426)^27^ and the complete proteome of pAO1 megaplasmid (GenBank: AJ507836.1)^10^. Additionally, Mascot was set up to search for contaminants in the common Repository of Adventitious Proteins database (2012.01.01; the Global Proteome Machine). Search parameters were strict trypsin specificity allowing for up to three missed cleavages sites, fragment ion mass tolerance of 1.30 Da and a parent ion tolerance of 0.8 Da. Oxidation of methionine was selected as variable modification and carbamidomethylation of Cys was specified as a fixed modification.

False positive levels were estimated via decoy database method with a reverse database appended at the end of the forward database^28^. Scaffold (v.4.8.2, Proteome Software Inc., Portland, OR, USA) was used to assemble the resulting database hits corresponding for the same biological data into one analysis using the MudPIT (Multidimensional Protein Identification Technology)^29^ and to validate MS/MS based peptide and protein identifications. The protein and peptide false discovery rate (FDR) was set to 1%. Protein identifications were accepted if the protein contained at least 2 identified peptides. Protein probabilities were assigned by the Protein Prophet algorithm^30^. Proteins that contained similar peptides and could not be differentiated based on MS/MS analysis alone were grouped to satisfy the principles of parsimony. Proteins sharing significant peptide evidence were grouped into clusters. All hits from the contaminants database were manually filtered out. Proteins were annotated with GO terms from Gene Ontology Consortium^31^.

Label-free relative quantification was performed using Scaffold (v.4.8.2, Proteome Software Inc., Portland, OR, USA) using weighted spectra and outputted as fold change. Significance was assessed using the Fisher’s Exact test and the p-values were further adjusted using the Benjamini and Hochberg correction to account for multiple comparisons^30^. An adjusted p-value of less than 0.05 was used to select proteins that were differentially expressed between compared groups. The mass spectrometry proteomics data have been deposited to the ProteomeXchange Consortium (http://proteomecentral.proteomexchange.org)^32^ via the MassIVE partner repository^33^ with the dataset identifier PXD008756.

## Electronic supplementary material

Supplementary information. Supplementary figure 1 and Supplementary figure 2

Supplementary table 1.

Supplementary table 2.

## Data Availability

The mass spectrometry proteomics data have been deposited to the ProteomeXchange Consortium (http://proteomecentral.proteomexchange.org) via the MassIVE partner repository with the dataset identifier PXD008756.
