# Supplementary material for: Proteomics based analysis of the nicotine catabolism in Paenarthrobacter nicotinovorans pAO1
Source: Sci Rep. 2018 Nov 2;8:16239. doi: 10.1038/s41598-018-34687-y (PMC6214936; doi:10.1038/s41598-018-34687-y)
Supplement: Supplementary file 1 — Supplementary information. Supplementary figure 1 and Supplementary figure 2 [file 41598_2018_34687_MOESM1_ESM.pdf]

Proteomics based analysis of the nicotine catabolism in *Paenarthrobacter nicotinovorans* pAO1

Marius Mihăşan[1,2*], Cornelia Babii[1], Roshanak Aslebagh[2], Devika Channaveerappa[2], Emmalyn Dupree[2], Costel C. Darie[2]

[1]Biochemistry and Molecular Biology Laboratory, Department of Biology, Alexandru Ioan Cuza University of Iaşi, Iaşi, Romania;

[2]Biochemistry & Proteomics Group, Department of Chemistry & Biomolecular Science, Clarkson University, Potsdam, NY, USA

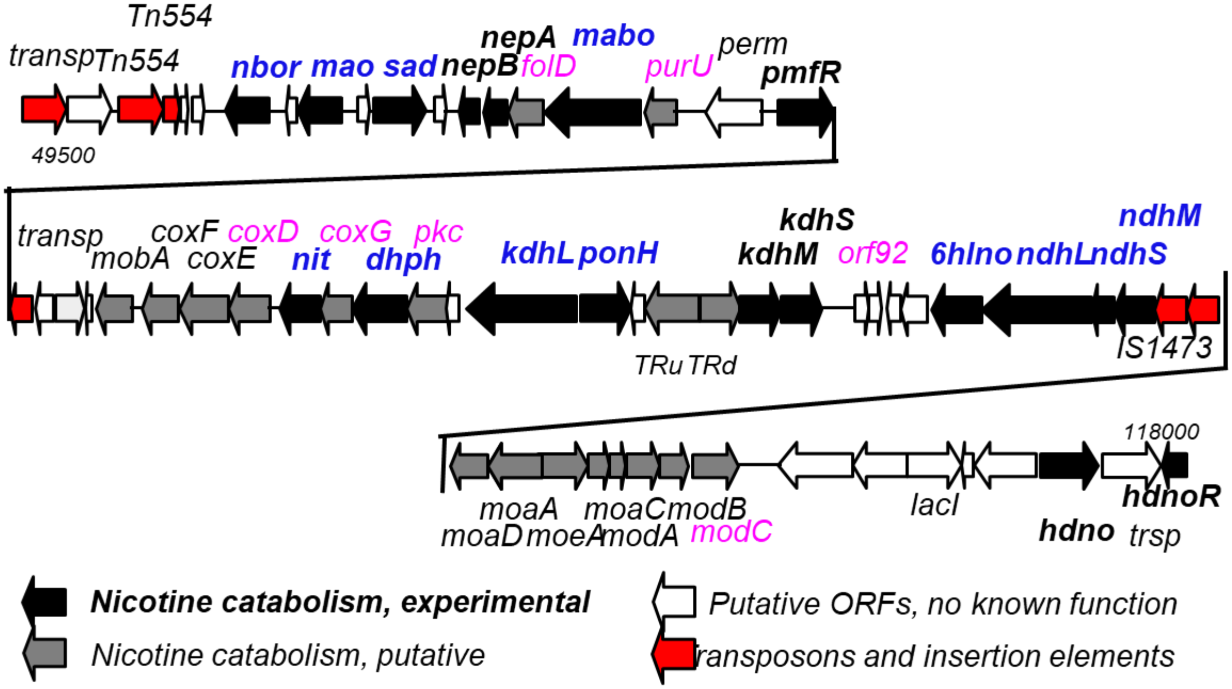

Supplementary figure 1. The *nic*-gene cluster on the pAO1 plasmid of *Paenarthrobacter nicotinovorans*. Blue text indicates genes with known function shown to have a nicotine-dependent expression by the proteomics experiment. Magenta text indicates genes with putative function related to nicotine and shown to have a nicotine-dependent expression by our proteomics experiment. **Black text** indicates genes that were not detected as expressed in our proteomics experiment.

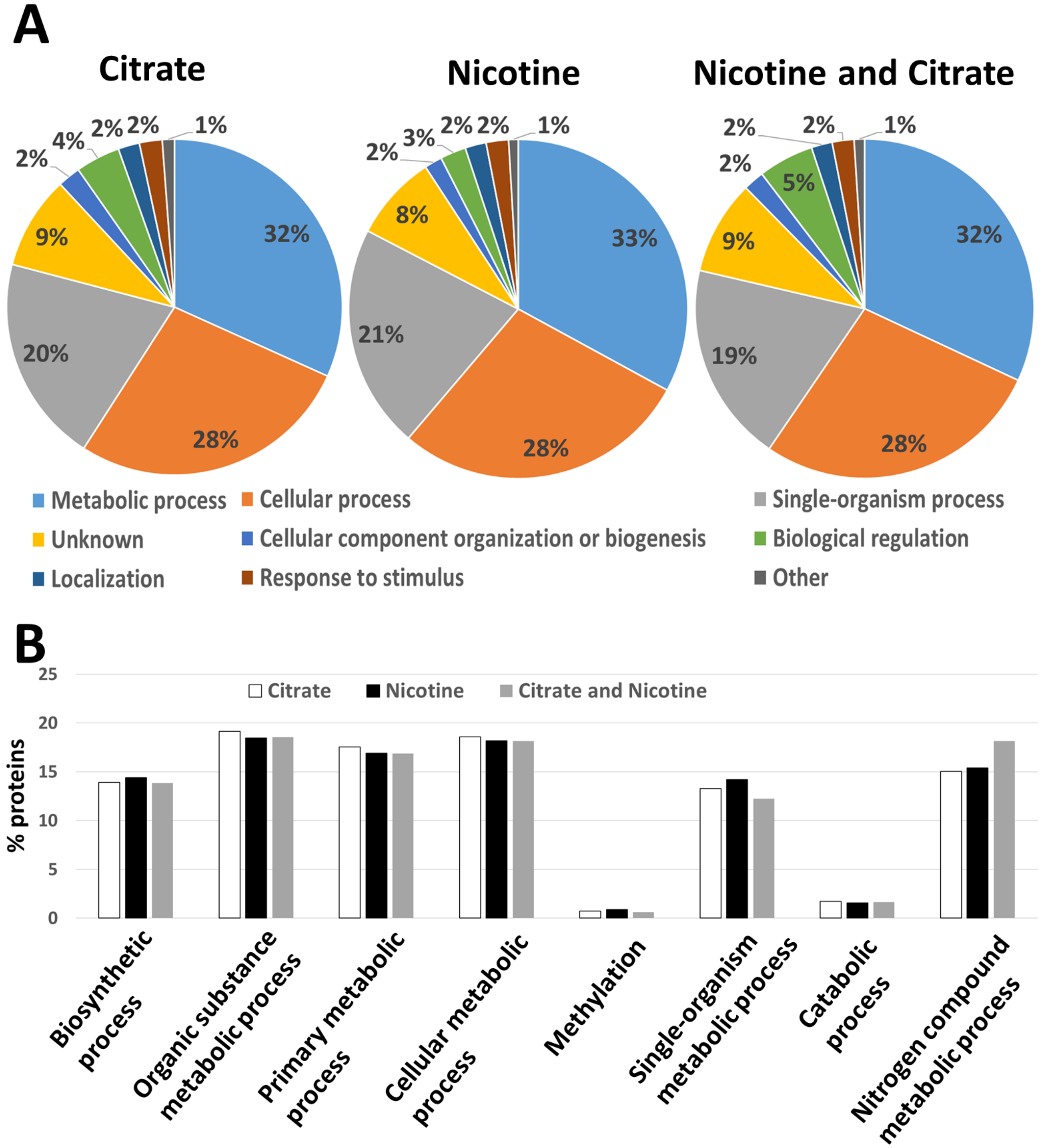

Supplementary figure 2. Grouping of the identified chromosomally-encoded proteins according to Gene Ontology Consortium using A. GO terms from the Biological process category and B. GO terms from the Metabolic process category.
